# Cholesterol-modifying drugs in COVID-19

**DOI:** 10.1093/oxfimm/iqaa001

**Published:** 2020-06-18

**Authors:** Nathalie M Schmidt, Peter A C Wing, Jane A McKeating, Mala K Maini

**Affiliations:** Division of Infection & Immunity, Institute of Immunity & Transplantation, University College London, London, UK; Nuffield Department of Medicine, Oxford University, Oxford, UK; Nuffield Department of Medicine, Oxford University, Oxford, UK; Division of Infection & Immunity, Institute of Immunity & Transplantation, University College London, London, UK

**Keywords:** COVID-19, SARS-CoV-2, viral immunology, T-cells, cholesterol-modifying drugs, inflammation, statins, dyslipidemia

## Abstract

Infection with severe acute respiratory syndrom coronavirus 2 (SARS-CoV-2) is more likely to lead to poor outcomes in the elderly and those with cardiovascular disease, obesity or metabolic syndrome. Here, we consider mechanisms by which dyslipidaemia and the use of cholesterol-modifying drugs could influence the virus–host relationship. Cholesterol is essential for the assembly, replication and infectivity of enveloped virus particles; we highlight several cholesterol-modifying drugs with the potential to alter the SARS-CoV-2 life cycle that could be tested in *in vitro* and *in vivo* models. Although cholesterol is an essential component of immune cell membranes, excess levels can dysregulate protective immunity and promote exaggerated pulmonary and systemic inflammatory responses. Statins block the production of multiple sterols, oxysterols and isoprenoids, resulting in a pleiotropic range of context-dependent effects on virus infectivity, immunity and inflammation. We highlight antiviral, immunomodulatory and anti-inflammatory effects of cholesterol-modifying drugs that merit further consideration in the management of SARS-CoV-2 infection.

## INTRODUCTION

The coronavirus disease 2019 (COVID-19) pandemic has highlighted the dynamic and variable nature of host–pathogen interactions, with the severity ranging from asymptomatic infection to fatal acute respiratory distress syndrome (ARDS). There is an urgent need to understand the mechanisms underlying the range of outcomes of severe acute respiratory syndrome coronavirus 2 (SARS-CoV-2) infection in order to consider repurposing existing drugs to limit morbidity and mortality. Poor outcomes are more frequent in older patients and in those with pre-existing cardiovascular disease, obesity and metabolic syndrome [1–3], implicating hyperlipidaemia as a possible exacerbating factor. The role of diabetes and antihypertensive drugs affecting the renin–angiotensin system (RAS) in these high-risk groups has been considered [4]. However, a sizeable proportion of those infected with SARS-CoV-2 is also taking statins, which are widely prescribed to reduce cardiovascular disease risk, with a good safety profile [5]. COVID-19 patients already taking statins were reported to have a milder course of infection in a care home setting [6]. Retrospective studies have previously found pre-existing statin usage to be associated with an improved outcome of pneumonia due to infections other than SARS-CoV-2 [7–9]. Statins are already being started in those not yet taking them during admission with COVID-19 in some centres [10]. Their use in SARS-CoV-2 infection has been proposed based on anecdotal effects in Ebola, although statin efficacy could not be distinguished from that of coadministered angiotensin II blockers, and randomized controlled trials were not performed [11].

Raised cholesterol or cholesterol-modifying drugs may be associated with differential rates of viral infection and outcome through indirect associations with other confounders and/or through direct effects. Here, we review existing mechanistic data that could provide a scientific rationale for hyperlipidaemia and cholesterol-modifying drugs playing a role in the course of SARS-CoV-2 infection. The initial disease outcome is likely to be determined by the balance between the efficiency of SARS-CoV-2 infectivity for tissues expressing the entry receptor angiotensin-converting enzyme 2 (ACE2) [12] (with resultant tissue cytopathology) and timely controlled antiviral immune responses. In contrast, the subsequent pathology seen in those COVID-19 patients progressing to ARDS is characterized by a cytokine storm accompanied by lung and systemic inflammation [13, 14]. We will consider three key categories by which cholesterol and cholesterol-modifying drugs could alter the outcome of SARS-CoV-2 infection: by modulating viral infectivity, antiviral immunity or inflammation.

## MODULATION OF SARS-CoV-2 INFECTION

Viruses have complex interactions with their host; cholesterol biosynthesis pathways are essential for the assembly, replication and infectivity of enveloped virus particles [15]. Cholesterol-modifying drugs could exert antiviral effects by altering target cell membrane cholesterol through reduced systemic absorption or synthesis or by direct antiviral activity. *In silico* prediction studies for drug repurposing suggested the potential for rosuvastatin, a member of the statin family, to bind to SARS-CoV-2 [16], whereas another study failed to report such an interaction [17]. A recent *in vitro* study investigating the SARS-CoV-2 protein–protein interactome identified scavenger receptor class B type 1 (SR-B1), a cholesterol trafficking receptor, as a potential drug target [18] and blockade of SR-BI inhibited SARS-CoV-2 infectivity in an *in vitro* study [19]. It is worth noting that SR-B1 is an essential cofactor for hepatitis C virus (HCV) infection and an SR-B1 inhibitor was well tolerated in an open-label clinical Phase Ib trial in HCV [20].

Lipid rafts, cholesterol-enriched microdomains in the cell membrane, play a role in coronavirus (CoV) entry into cells [21–23] and specifically in the activity of SARS-CoV fusion peptide [24]. Increasing total cellular cholesterol levels promoted ACE2 recruitment to lipid rafts and enhanced SARS-CoV-2 pseudovirus entry [25]. Conversely, Takano *et al.* showed a role for the cholesterol transport inhibitor U18666A in reducing feline CoV infection [26]. U18666A inhibited both cholesterol biosynthesis and functional activity of the cholesterol transporter Niemann–Pick C1 (NPC1), a receptor that has been implicated in the life cycle of several viruses [26–28], highlighting a need for further studies to evaluate the role of NPC1 in SARS-CoV-2 infection. A recent screen of existing Food and Drug Administration (FDA)-approved drugs for potential activity against SARS-CoV-2 identified cepharanthine as a potent inhibitor of virus–cell attachment [29], with a previous report suggesting it has broad antiviral activity against CoV [30]. This natural compound targets several aspects of cell metabolism including cholesterol trafficking [31]. Statins reduce intracellular and extracellular cholesterol by targeting 3-hydroxy-3-methylglutaryl-Coenzyme A (HMG-CoA) reductase, the rate-limiting first step in the cholesterol biosynthesis (mevalonate) pathway; they therefore also inhibit the synthesis of multiple other biologically active sterols, oxysterols and key isoprenoid intermediates [32, 33]. Their inhibition of protein prenylation via the reduction of isoprenoid intermediates could provide an additional mechanism to inhibit SARS-CoV-2 infectivity (reviewed by Marakasova *et al*. [34]).

Much of our current knowledge on coronavirus biology was acquired using mouse hepatitis virus (MHV) as a model system. Early studies on the viral encoded structural proteins showed that the MHV spike protein (S) is synthesized as a precursor and intracellular proteolytic cleavage events expose the fusion peptide necessary for viral entry into cells [35]. The SARS-CoV-2 spike protein encodes a furin cleavage site in the S1/S2 junction that differs from SARS-CoV and other closely related bat viruses [36], which may influence particle infectivity. The SARS-CoV-2 spike protein shows a 10- to 20-fold higher affinity for ACE2 than SARS-CoV, which may explain its higher transmissibility [12]. Statins were reported to increase cardiac ACE2 expression in a rabbit model of atherosclerosis [37]; however, further reports in rodent models show a negligible effect of statins on ACE2 expression levels [38, 39]. It will be important to evaluate the impact of statins on ACE2 expression in human lung epithelia as this may impact on viral entry and enhance tissue tropism in early infection (while also promoting anti-inflammatory effects through ACE2 discussed below). However, it is worth noting that coronavirus tropism is not solely defined by the spike protein, with the nucleocapsid, replicase and accessory genes also playing a role (reviewed by Weiss and Leibowitz [40]). Hence one should take care to infer viral pathogenesis from spike protein–ACE2 affinities alone and await further studies on SARS-CoV-2 tissue tropism.

## MODULATION OF ANTIVIRAL IMMUNITY

Distinguishing protective from pathogenic immune responses to SARS-CoV-2 will aid the development of successful vaccines and other immunotherapeutic approaches; it will also allow the identification of biomarkers to detect patients mounting effective responses that limit the severity of natural infection. Although these immune correlates are still being defined for SARS-CoV-2, we can speculate how cholesterol may impact on protective immune responses, based on related studies. The complex effects of sterols, and the drugs modulating them, on different components of the immune system have been reviewed previously [33, 41–43]; here we will highlight some relevant examples.

Cholesterol is an essential component of cell membranes and lipid rafts that facilitate immune synapse formation and downstream signalling [44–46]. Intracellular levels of cholesterol are tightly regulated, with the transcription factors liver X receptor (LXR) and sterol regulatory element-binding protein (SREBP) serving as metabolic checkpoints regulating the shift between resting and proliferating T cells [47, 48]. However, cholesterol metabolism and the mevalonate pathway are not only important for effector cells but can potently drive the proliferation of regulatory T cells, enhancing and stabilizing their suppressive capacity [49, 50]. Mice fed a high cholesterol diet upregulate the inhibitory ligand PD-L1 on B cells, which can suppress follicular helper T-cell differentiation to limit the adaptive response [51]. High cholesterol in the tumour environment can suppress CD8^+^ T cells, promoting the transcription of exhaustion markers like PD-1 through the ER stress sensor X-box-binding protein 1 (XBP1) [52] and inhibiting interleukin-9 (IL-9) expression through LXR sumoylation [53]. Esterified cholesterol accumulates in neutral lipid droplets, an excess of which can be cytotoxic and limit immune cell functionality (as exemplified by natural killer cells [54]). Thus, several lines of evidence suggest that disrupted cholesterol homeostasis could alter the balance of effector and inhibitory immune responses to limit effective antiviral control.

By blocking cholesterol synthesis, statins may be able to reverse some of the immunosuppressive effects driven by excess cholesterol described above, to promote functional antiviral immune responses [32, 33, 52]. In addition, a number of studies have underscored the immunomodulatory potential of statins beyond their lipid-lowering capacity [32]. By blocking HMG-CoA reductase, statins also reduce synthesis of a number of other relevant mediators including oxysterols, that have multiple immunomodulatory roles [33] and 25-hydroxycholesterol, involved in the Type I interferon (IFN) antiviral response [55]. The statin-induced block in 25-hydroxycholesterol synthesis has also been shown to limit the switching of human CD4^+^T cells from production of the antiviral/pro-inflammatory cytokine IFN-γ to the immunoregulatory IL-10 [56], suggesting they could enhance the antiviral capability of CD4^+^ T cells while limiting their suppressive potential.

A mechanism underlying several of the immunomodulatory effects of statins is the reduced isoprenylation of small GTPases [57, 58]. Of particular relevance to the quest for a vaccine to protect against SARS-CoV-2, lipophilic statins can act as vaccine adjuvants in animal models by inhibiting prenylation of Rab5a in antigen-presenting cells; this arrested endosomal maturation and enhanced antigen retention for presentation, thereby boosting germinal centre B-cell responses for high-affinity antibodies as well as improving CD8^+^ T-cell responses [59]. The adjuvant potential of statins was supported by a small placebo-controlled prospective interventional study showing increased antibody titres to pneumococcal vaccines in healthy subjects given the lipophilic atorvastatin [60]. However, observational studies in elderly people taking long-term statins have shown mixed effects on vaccine responses to influenza [61], raising the possibility that their adjuvant capacity is lost with chronic use.

## MODULATION OF INFLAMMATION

There is a delicate balance between the protective antiviral immune responses considered above and uncontrolled bystander responses that drive the cytokine storm and inflammation characterizing the ARDS phase of SARS-CoV-2 infection. Since cholesterol has been reported to induce inflammatory signalling in macrophages and CD11c+ cells [42, 62], pre-existing hypercholesterolaemia may alter the pathological response to SARS-CoV-2. Oxidized low-density lipoprotein (LDL) and cholesterol crystals can activate NOD-, LRR- and pyrin domain-containing protein 3 (NLRP3) inflammasomes to activate IL-1β and IL-18 production [63]. Accumulation of unesterified cholesterol in macrophages is associated with the production of the pro-inflammatory cytokines tumour necrosis factor-alpha (TNF-α) and IL-6 [64] that early data suggest are a feature of SARS-CoV-2 immunopathogenesis [13, 14]. Interestingly, cholesterol constitutes the major neutral lipid in lung surfactant, raising the possibility that hypercholesterolaemia can dysregulate the protective properties of surfactant in alveolar spaces [65]. Lipid-laden macrophages (foam cells), a common feature in lung pathology, are more likely to accumulate in hypercholesterolaemia [65] and would be expected to predispose to an exaggerated inflammatory response to SARS-CoV-2.

Statins have the potential to limit inflammation by altering a number of mediators from the cholesterol biosynthesis pathway. These anti-inflammatory properties are thought to be central to their protective effects in cardiovascular disease beyond the simple reduction in cholesterol [32]. The many other ways in which statins can exert anti-inflammatory effects include their capacity to stabilize endothelial leakage, limit leukocyte transmigration and increase local nitric oxide [32]. These are likely to be relevant to the pathology of SARS-CoV-2, which can include endothelial cell infection and endotheliitis in several organs [66]. If the published data suggesting that statins can increase the expression of ACE2 in animal models [37] hold out *in vivo* in humans, their impact on the RAS axis would provide a further mechanism by which they could exert an anti-inflammatory effect, with ACE2 converting the pro-inflammatory angiotensin II into angiotensin (1–7), which act on the Mas receptor to attenuate inflammation through nitric oxide [67].

However, the pleiotropic effects of statins make it difficult for *in vitro* and *in vivo* models to predict the full complexity of their action in different disease states in humans. For example, an intervention study of rosuvastatin in healthy normocholesterolaemic subjects resulted in a small increase in circulating inflammatory cytokines [68], whereas pravastatin treatment of subjects with hypercholesterolaemia reduced their production of IL-6 and TNF-α following *in vitro* stimulation with lipopolysaccharide (LPS) [69]. Of note, data from several large randomized trials testing the addition of statins for ARDS [70–72] (of non-SARS-CoV-2 aetiologies) showed no overall benefit or capacity to combat rising levels of IL-18 [73], suggesting they are unlikely to exert useful anti-inflammatory activity if started in the advanced stages of COVID-19.

## CONCLUSIONS

Several lines of evidence provide a rationale for high cholesterol predisposing to a worse outcome in SARS-CoV-2 infection, by dysregulating protective immunity and promoting exaggerated pulmonary and systemic inflammatory responses. The capacity of statins to reduce lipids, boost protective immune responses and exert anti-inflammatory properties could all contribute to a potential benefit during SARS-CoV-2 infection.

However, further studies are required to fully understand the impact of hyperlipidaemia and cholesterol-modifying drugs on the clinical course of SARS-CoV-2 infection, with careful adjustment for potential confounders. Statin compliance, shown to be very low in some studies [74], should ideally be assessed and the use of lipophilic and hydrophilic statins examined separately since their differential cell permeability can alter their mechanisms of action [43]. Genetic polymorphisms influencing cholesterol uptake and efflux transporters may also need to be considered [75]. Of particular note, the acute phase response in severe viral infections can be accompanied by transient dyslipidaemia [76] that may not accurately reflect pre-illness levels. This is exemplified by studies in hospitalized patients with SARS-CoV-2, revealing striking reductions in plasma LDL and high-density lipoprotein (HDL) levels, that were associated with disease severity and only returned to the normal range 2 weeks after recovery (in one patient who was followed longitudinally) [77, 78]. Whether these transient reductions and changes in oxidized lipoproteins contribute to disease pathogenesis, as suggested in HIV [79], remains to be investigated. However, these data imply that reliable assessment of associations between dyslipidaemia and SARS-CoV-2 outcome will require data on lipid levels measured prior to, as well as during, the illness.

In conclusion, carefully conducted analysis of available patient data will contribute to further assessment of the relevance of dyslipidaemia and recommendations on the future use of cholesterol-modifying drugs in the setting of SARS-CoV-2. The therapeutic potential of statins across the spectrum, from vaccine trials in healthy individuals to early and advanced phases of SARS-CoV-2, needs further evaluation. We have highlighted several other cholesterol-modifying drugs of potential relevance to SARS-CoV-2 outcome. These, along with statins, merit testing in primary human epithelial cultures and animal models replicating SARS-CoV-2 for their potential range of antiviral, immunomodulatory and anti-inflammatory effects.

## FUNDING

Work in the authors’ laboratories is funded by a Wellcome Trust Investigator Award (101849/Z/13/A) to M.K.M., Cancer Research UK project grant (26603) to M.K.M., Cancer Research UK Hepatocellular Carcinoma Expediter Network to M.K.M., a Wellcome Trust Investigator Award (200838/Z/16/Z) to J.A.M. and an Medical Research Council project grant (MR/R022011/1) to J.A.M.

## AUTHORS’ CONTRIBUTION

All authors carried out literature searches, reviewed published work and contributed to the writing of the manuscript. All authors checked and approved the final version.

## CONFLICT OF INTEREST STATEMENT

The authors hold a pending UK priority patent application No.1917498.6 entitled ‘Treatment of Hepatitis B Virus (HBV) Infection’. Unrelated to the content of this manuscript, the Maini Lab has received unrestricted funding from Gilead, Roche and Immunocore. M.K.M. has sat on advisory boards/provided consultancy for Gilead, Hoffmann La Roche, Immunocore, VIR, Galapagos NV, GSK, Abbvie and Freeline.

## DATA AVAILABILITY

No new data were generated or analysed in support of this research.

## Supplementary Material

iqaa001_Supplementary_Data
