# Supplementary material for: Cholesterol-modifying drugs in COVID-19
Source: Oxf Open Immunol. 2020 Jun 18;1(1):iqaa001. doi: 10.1093/oxfimm/iqaa001 (PMC7337782; doi:10.1093/oxfimm/iqaa001)
Supplement: iqaa001_Supplementary_Data [file iqaa001_supplementary_data.zip › Report 1.docx]

**Report Referee 1**

Comments to the Author:-

This is an excellent manuscript highlighting not only important aspects of CoV-2 infection biology but also pointing the direction to targeted therapeutic interventions. It is a pleasure to read and is highly timely.
