# Supplementary material for: Cholesterol-modifying drugs in COVID-19
Source: Oxf Open Immunol. 2020 Jun 18;1(1):iqaa001. doi: 10.1093/oxfimm/iqaa001 (PMC7337782; doi:10.1093/oxfimm/iqaa001)
Supplement: iqaa001_Supplementary_Data [file iqaa001_supplementary_data.zip › Report 2.docx]

**Report Referee 2**

Comments to the Author:-

Schmidt and colleagues have written a timely review about the therapeutic potential of cholesterol-modifying drugs in COVID-19. It is well written and makes interesting points that may stimulate interest and debate in relation to the therapeutic landscape insofar as they have appraised the antiviral, immune modulatory and anti-inflammatory potential of cholesterol modifying drugs. It might benefit from a schematic?
